# Impact of Host DNA and Sequencing Depth on the Taxonomic Resolution of Whole Metagenome Sequencing for Microbiome Analysis

**DOI:** 10.3389/fmicb.2019.01277

**Published:** 2019-06-12

**Authors:** Joana Pereira-Marques, Anne Hout, Rui M. Ferreira, Michiel Weber, Ines Pinto-Ribeiro, Leen-Jan van Doorn, Cornelis Willem Knetsch, Ceu Figueiredo

**Affiliations:** ^1^i3S – Instituto de Investigação e Inovação em Saúde, Universidade do Porto, Porto, Portugal; ^2^Ipatimup – Instituto de Patologia e Imunologia Molecular da Universidade do Porto, Porto, Portugal; ^3^Instituto de Ciências Biomédicas Abel Salazar da Universidade do Porto, Porto, Portugal; ^4^DDL Diagnostic Laboratory, Rijswijk, Netherlands; ^5^Departmento de Patologia, Faculdade de Medicina da Universidade do Porto, Porto, Portugal

**Keywords:** microbiome analysis, metagenomics, sample complexity, sequencing depth, mock community

## Abstract

The amount of host DNA poses a major challenge to metagenome analysis. However, there is no guidance on the levels of host DNA, nor on the depth of sequencing needed to acquire meaningful information from whole metagenome sequencing (WMS). Here, we evaluated the impact of a wide range of amounts of host DNA and sequencing depths on microbiome taxonomic profiling using WMS. Synthetic samples with increasing levels of host DNA were created by spiking DNA of a mock bacterial community, with DNA from a mouse-derived cell line. Taxonomic analysis revealed that increasing proportions of host DNA led to decreased sensitivity in detecting very low and low abundant species. Reduction of sequencing depth had major impact on the sensitivity of WMS for profiling samples with 90% host DNA, increasing the number of undetected species. Finally, analysis of simulated datasets with fixed depth of 10 million reads confirmed that microbiome profiling becomes more inaccurate as the level of host DNA increases in a sample. In conclusion, samples with high amounts of host DNA coupled with reduced sequencing depths, decrease WMS coverage for characterization of the microbiome. This study highlights the importance of carefully considering these aspects in the design of WMS experiments to maximize microbiome analyses.

## Introduction

The collection of microorganisms present in a defined environment is known as the microbiota (Marchesi and Ravel, 2015). These microorganisms, which comprise bacteria, archaea, viruses, and microbial eukaryotes, along with their genetic information and specific characteristics of the niche they occupy are generally known as the microbiome (Marchesi and Ravel, 2015).

Targeted 16S rRNA gene and whole metagenome sequencing (WMS) are sequencing-based approaches that are currently used to explore the composition and functions of the microbiome (Human Microbiome Jumpstart Reference Strains Consortium, et al., 2010; Human Microbiome Project Consortium, 2012a). WMS consists on untargeted DNA sequencing of fragments of all genomes within a sample, which produces high-complexity datasets with millions of short reads (Quince et al., 2017). Since all DNA present in the sample is captured, including bacterial, viral, and eukaryotic DNA, this method allows extensive characterization of the microbial communities living in a wide range of environments (e.g., soil, human-associated samples, among others) (Venter et al., 2004; Qin et al., 2010). In comparison with targeted 16S rRNA sequencing, WMS typically yields a more detailed taxonomic resolution, at the species or even strain-level. It also provides a more accurate insight into the functional composition of the microbiome (Qin et al., 2010; Abubucker et al., 2012; Truong et al., 2015; Truong et al., 2017). Still, this approach has been less implemented since it is more expensive than 16S rRNA profiling, it requires a greater depth of coverage, and the data analysis is more complex (Knight et al., 2012).

It is currently well recognized that the microbiome plays an important role in human physiology and in the maintenance of health, but also has a major impact in the development of a wide range of diseases, including obesity, inflammatory bowel disease, and cancer (Ley et al., 2006; Frank et al., 2007; Kostic et al., 2013; Gilbert et al., 2018). A major technical challenge in whole metagenome analysis of human samples is the predominance of host DNA. Data from the Human Microbiome Project (HMP) has revealed that the proportion of human DNA differs significantly by body site and sample type (Human Microbiome Project Consortium, 2012b; Lloyd-Price et al., 2017). While stool samples comprise less than 10% of human DNA, samples such as saliva, throat, buccal mucosa, and vaginal swabs contain more than 90% of human-aligned reads (Human Microbiome Project Consortium, 2012b; Lloyd-Price et al., 2017). The latter type of samples, where only a limited fraction of the DNA represents the microbial content, requires a high quantity of sequences to obtain a reasonable coverage of the microbial genomes when using WMS. Currently, very little is known about the impact of this technical limitation on the sensitivity of WMS to profile the microbiome of host-derived samples. In addition, there is no guidance for the reasonable amount of host DNA a sample should contain in order to generate an accurate WMS analysis. Overcoming these issues is crucial for future selection of appropriate sequencing depths that will guarantee the return of the maximum useful information, with a minimum cost possible. Therefore, this study aimed to evaluate the sensitivity of WMS for taxonomic profiling of microbiome samples, taking into account the wide range of host DNA in a sample and sequencing depths.

## Materials and Methods

### Mock Microbial Community

Genomic DNA from Microbial Mock Community B (Staggered, High Concentration), v5.2H, for Whole Genome Shotgun Sequencing, HM-277D, was obtained through BEI Resources, NIAID, NIH as part of the HMP. This mock microbial community is composed of a combination of 20 bacterial genomic DNAs that differ in %GC content (30 to 69%), and contains staggered ribosomal RNA operon counts differing by bacteria, ranging from 10^4^ to 10^7^ copies per organism per μL (as indicated by the manufacturer). The genomic GC content of each species was obtained from the NCBI Genome Database. To estimate the expected relative abundance of species, the theoretical number of genome copies per species was calculated by the ratio of input 16S rRNA copies to 16S rRNA copies per genome, and normalized by the sum of all theoretical genome copies of the species present in the mock (sum up to 100). The detailed composition of the mock community, including %GC content, the number of 16S rRNA copies per genome, the number of 16S rRNA input copies, the number of species genome copies, and the expected relative abundance of species, is available in the Supplementary Table S1.

### Mouse Cell Line and DNA Isolation

Total genomic DNA was extracted from the MC-38 cell line (a kind gift from Professor J. Machado, University of Porto), which is derived from C57BL/6 murine colon adenocarcinoma cells, with the QIAamp DNA Tissue kit (Qiagen, Germany), according to the manufacturer’s instructions. DNA was eluted in 100 μl Microbial-DNA free water (Qiagen).

### Generation of Synthetic Samples

To create different synthetic samples (SS) with well-defined ratios of host to bacterial DNA, the mock microbial community DNA was spiked with DNA from the mouse cell line. DNA concentrations of the mock microbial community and of the mouse cell line were measured using the NanoDrop 2000 UV spectrophotometer (Thermo Fisher Scientific), and the exact volumes to be mixed in each condition were determined. SS with increasing ratios of host to bacterial DNA were generated containing 10% (SS10), 90% (SS90), and 99% (SS99) host DNA. The mock microbial community sample (MS), which contains only microbial DNA, was used as control.

### Library Preparation and Whole Metagenome Sequencing

Samples were first quantified and normalized to 0.2 ng/μl DNA material, using a Quant-It PicoGreen dsDNA assay (Thermo Fisher Scientific), in order to use 1 ng input DNA for the library construction. Metagenomic library preparation was automated on the Hamilton Microlab STAR Liquid Handling Workstation, using a Nextera XT DNA library preparation kit (Illumina Inc., CA, United States) according to the manufacturer’s protocol. Briefly, after normalized samples were fragmented and tagged by tagmentation, a limited-cycle PCR was performed to add the Index 1 (i7), Index 2 (i5) and full adapter sequences required for cluster generation. Amplification was followed by a cleanup step that purified the library DNA and removed small library fragments by using Agencourt AMPure XP beads (Beckman Coulter, Inc.). The quality of the library was assessed using an Agilent Technology 2100 Bioanalyzer (Agilent Technologies, Wokingham, United Kingdom) and then, a bead-based normalization was performed using beads Nextera XT to ensure more equal library representation in the pooled library. Finally, the pooled library was sequenced as a paired-end 150-cycle run on the Illumina NextSeq 550 platform, at an expected sequencing depth of 5.5 Gb/sample.

### Sequencing Data Analysis

For each sample, the two FASTQ files with the forward and reverse paired-end reads were concatenated into one single FASTQ file, which was used as input for our in-house pipeline of WMS sequencing data analysis.

#### Sequencing Data Pre-processing

Sequencing data pre-processing was performed by KneadData (version 0.6.1), a computational tool designed to perform quality control on metagenomic sequencing data. KneadData integrates the tools FastQC (version 0.11.5) (Andrews, 2016), Trimmomatic (version 0.33) (Bolger et al., 2014), and Bowtie2 (version 2.2) (Langmead and Salzberg, 2012), to do quality check, quality filtering, and host sequences decontamination, respectively.

First, reads were trimmed based on a sliding window trimming approach, cutting once the average base Phred quality score within a four-base sliding window dropped below 20, and then were discarded when the length of the read was shorter than 60 bp. After the quality-filtering step, KneadData used Bowtie2 to identify and remove the mouse contaminant reads present in the datasets, by mapping the reads against the C57BL/6 reference genome (GCA_001632555.1 assembly). The non-mouse filtered reads were then used for the downstream analysis. Bowtie2 was used with the default parameters (–very-sensitive end-to-end alignment). FastQC, as a component of KneadData, performed quality control checks on raw whole metagenome sequencing data but also on reads after sequencing data pre-processing, in order to assess the efficiency of the quality filtering and of host sequences decontamination steps in the generation of high-quality reads. FastQC was used with the default parameters (Andrews, 2016).

#### Taxonomic Profiling – MetaPhlAn2

In our in-house pipeline of analysis, the host-filtered microbial reads were taxonomically profiled using MetaPhlAn2 (version 2.7.5), an assembly free taxonomic profiler (Segata et al., 2012; Truong et al., 2015). This computational tool mapped the quality-controlled shotgun reads to a database of unique clade-specific marker genes (read-based profiling) with high discriminatory power, estimating the relative abundances of each microbial clade in the samples with species-level resolution (Segata et al., 2012; Truong et al., 2015). Bowtie2, a fast DNA aligner, is used by MetaPhlAn2 to map the metagenomic reads against the unique clade-specific marker genes. Clade-specific markers constitute coding sequences that unambiguously identify specific microbial clades (at different taxonomic levels). MetaPhlAn2 relies on ∼1 million unique clade-specific marker genes identified from ∼17,000 reference genomes and >7,000 unique species. Markers are now identified not only for Bacteria and *Archaea* (∼13,500 bacterial and archaeal genomes), but also for Viruses (∼3,500 viral genomes) and Eukaryotic microorganisms (Fungi and Protozoa; ∼110 eukaryotic genome (Truong et al., 2015).

### Generation of Datasets With Reduced Sequencing Depths

The sample with the largest sequence dataset (SS90) comprising 50.8 million single-end reads and a high predominance of host DNA was used. Four datasets with reduced sequencing depths were generated by random subsampling paired-end reads using an in-house script. From the original SS90 dataset, we subsampled 50, 25, 10, and 5%, which correspond to 25.4, 12.7, 5.1, and 2.5 million single-end reads, respectively. For subsampling, the same random seed was used in order to guarantee that the reads from the same pair were subsampled in the forward and reverse FASTQs. Then, it was created a new set of paired FASTQ file for each random subset. At each depth, the subsampling analysis was repeated five times.

### Generation of Simulated Datasets of Microbiome Samples With Different Host-Microbial Ratios

Simulated datasets (SD) of microbiome samples with different host: microbial ratios were created by randomly selecting host and microbial reads from our previously sequenced datasets, and combining them in different proportions at a fixed sequencing depth of 10 million single-end reads, using an in-house script. Microbial single-end reads were randomly picked from the MS raw dataset, to assure that only microbial reads were selected. Host single-end reads were randomly picked from the mouse contaminant sequences removed by KneadData from the SS99 raw dataset, to guarantee sufficient sequences with host origin (the raw SS99 dataset contained 33.201.587 mouse single-end reads). Eighteen SD were generated, nine with progressive 10% increases in host reads (SD10 to SD90) and nine with progressive 1% increases in host reads (SD91 to SD99). For each simulated dataset, five replicates were randomly generated.

### Statistical Analyses

Statistical treatment was performed using the GraphPad Prism software (v. 6.01, GraphPad Software Inc., La Jolla, CA, United States). Pearson’s correlation was used to assess correlations between the species genomic %GC content and the ratio between observed and expected relative abundances in the MS control. A ≥2-fold difference was selected as arbitrary threshold to consider species as underestimated or overestimated in comparison with a reference condition. Differences between groups, when performing the random subsampling analysis and the simulated dataset analysis, were evaluated using the Kruskal–Wallis non-parametric test, followed by multiple comparisons versus a control group using the Dunn’s test. The differences were considered statistically significant with *P* values lower than 0.05.

## Results

### Generation of Synthetic Samples and Pre-processing of Sequencing Data

**FIGURE 1:**
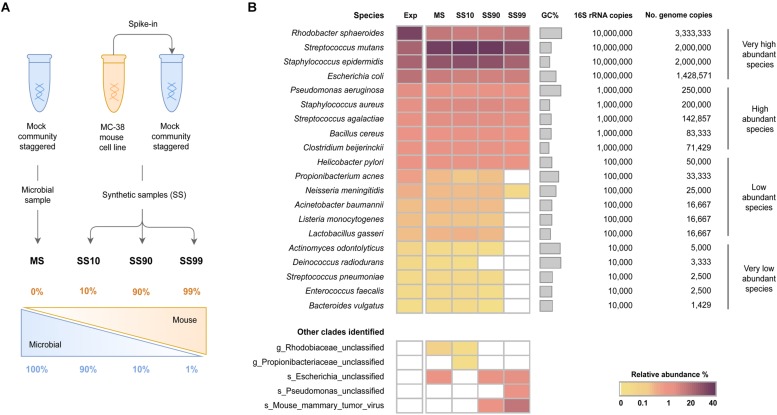
Effect of the levels of host DNA on the sensitivity of WMS for microbiome taxonomic profiling. **(A)** Schematic representation of the experimental design to generate synthetic samples. DNA samples from a mock microbial community staggered (HM-277D) were spiked with DNA from a mouse cell line (MC-38 cells), generating three synthetic samples containing 10, 90, and 99% host DNA (SS10, SS90, and SS99, respectively). DNA from the mock microbial community was used as control (MS). **(B)** Taxonomic profile of the metagenomes of the synthetic samples determined with MetaPhlAn2, and expressed as relative abundance of species in a heat map. The expected (Exp) taxonomic results were estimated based on the theoretical number of species genome copies present in the mock. Species were sorted from the highest to the lowest expected relative abundances.

To assess the influence of host DNA on the sensitivity of WMS for taxonomic profiling of the microbiome, three synthetic samples with distinct host: bacteria DNA ratios were generated to contain 10, 90, and 99% host DNA (SS10, SS90, and SS99, respectively). As control, the mock microbial community DNA sample (100% bacterial DNA; MS) was used (Figure 1A).

The four datasets yielded a large number of raw single-end reads ranging from 35 to 51 million. After sequencing data pre-processing (quality filtering and host sequences decontamination), the number of reads differed considerably between samples, being higher in MS (33.3 million reads) and SS10 (29.9 million reads) in comparison with SS90 (5.5 million reads) and SS99 (7.5 hundred thousand reads). The relative low number of pre-processed reads in SS90 and SS99 samples was due to high number of host DNA sequences removed rather than to reads dropped during quality filtering (Supplementary Table S2).

Of the total raw single-end reads, the proportion of discarded reads during quality filtering was similar between all samples (ranging from 16 to 19%), confirming that differences in the number of pre-processed reads across samples were associated with the host sequences decontamination step (Supplementary Figure S1A). These results are consistent with the overall good quality of all raw datasets (between 80 and 90% of the reads with average quality ≥Q30). They also indicate that quality filtering was appropriate, resulting in datasets with reads of extremely high quality (99% of the reads had an average quality ≥Q30; Supplementary Figures S1B,C). Quality-filtered reads were comparable with the expected ratios of host to microbial DNA for each condition (Supplementary Figure S1A).

Overall, synthetic samples with the expected host to bacterial DNA ratios were successfully generated.

### Effect of the Level of Host DNA on the Sensitivity of WMS for Microbiome Taxonomic Profiling

After sequencing data pre-processing, the taxonomic profile of all samples was determined with MetaPhlAn2, with the aim to evaluate the effect of host DNA on the sensitivity of WMS for microbiome profiling. For that, bacteria species were grouped into the following categories, according to the number of 16S rRNA copies in the mock community: very low (10^4^), low (10^5^), high (10^6^), and very high (10^7^) abundant. The relative abundance of each taxa was then quantified at species-level and represented in a heat map (Figure 1B).

In the control MS, all 20 species of bacteria were successfully identified with a similar taxonomic profile compared to that of the expected, calculated based on the theoretical number of genome copies (Figure 1B). In three species, there was over- or underestimation of the relative abundances due to the GC content bias introduced during Illumina sequencing (Supplementary Table S1 and Supplementary Figure S2).

The microbial profile of SS10 was comparable to the MS control, since all 20 bacterial species were detected with similar relative abundances to those of the MS (Figure 1B and Supplementary Table S3). In SS90, however, there was a decrease in the ability to detect very low abundant species. Specifically, *Deinococcus radiodurans* could not be identified (Figure 1B), and the relative abundances of *Actinomyces odontolyticus*, *Enterococcus faecalis*, and *Bacteroides vulgatus* were underestimated (Supplementary Table S3). The reduction in sensitivity was more striking in SS99, where only two of the low abundant and none of the very low abundant species were identified (Figure 1B).

In all conditions, unclassified clades were identified at low relative abundances (<2%), which likely represent bacterial species from the mock microbial community that were identified only at the genus or family level. Also, in synthetic samples with the highest amount of host DNA (SS90 and SS99), a mouse mammary tumor virus was identified (Figure 1B). Since viruses are not included in the mock community, the virus was likely introduced in the generation of the synthetic samples by the spiking with DNA from the mouse cell line, which could have the virus integrated in its genome. Besides, viruses are not included in the database used by Bowtie2, and therefore viral sequences have not been filtered as host contaminant.

These results show that the taxonomic profile of the mock microbial community was accurately reconstituted. Results also demonstrate that high ratios of host: bacterial DNA interfere with the sensitivity of WMS for taxonomic profiling. The increase in the proportion of host DNA leads to decreased sensitivity of WMS to detect very low and low abundant bacterial species.

### Impact of Sequencing Depth on the Sensitivity of WMS for Microbiome Taxonomic Profiling

**FIGURE 2:**
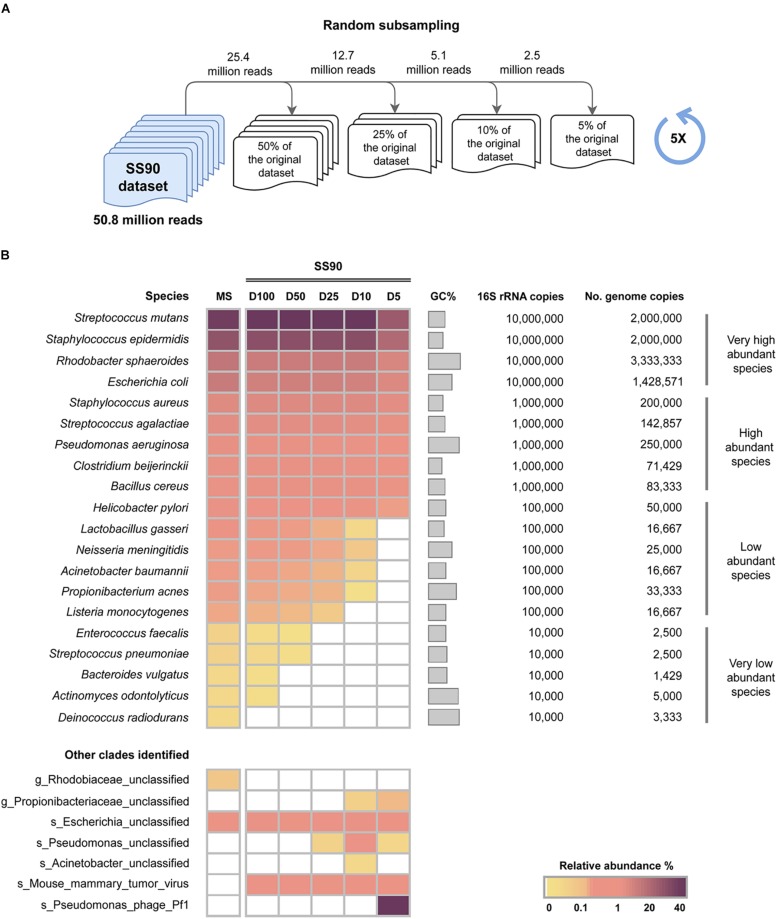
Impact of sequencing depth on the sensitivity of WMS for microbiome taxonomic profiling. **(A)** Schematic representation of the experimental design to generate random subsampling reads from the SS90 original dataset (90% host DNA). Random subsampling corresponding to 50, 25, 10, and 5% of the reads from the original dataset (SS90D50, SS90D25, SS90D10, and SS90D5, respectively) were generated. **(B)** Taxonomic profile of the generated datasets are represented as the average relative abundance from five independent experiments, and shown in a heat map. Data was sorted from the highest to the lowest relative abundances of species in the mock microbial community (MS).

To assess the impact of sequencing depth on the sensitivity of WMS to detect bacterial species in samples with a high level of host DNA, reads from the SS90 metagenome were randomly subsampled, generating four datasets with reduced sequencing depths, corresponding to 50, 25, 10, and 5% of the original dataset (SS90D50, SS90D25, SS90D10, and SS90D5, respectively). Their taxonomic profile was compared to that of the original SS90 dataset (SS90D100; Figure 2A).

When the SS90 dataset was reduced to half of its original size (SS90D50), the number of very low abundant species that were not identified increased from one to three (Figure 2B), and the relative abundance of *E. faecalis* significantly decreased (*P* = 0.006; Supplementary Tables S4, S5). In SS90D25, none of the very low abundant species could be identified (Figure 2B). In comparison with the original dataset, no statistically significant differences were observed in the relative abundances of the remaining species (Supplementary Tables S4, S5). In SS90D10 and SS90D5, however, in addition to not identifying all very low abundant species, there were statistically significant decreases in the relative abundances of the low abundant species (Figure 2B and Supplementary Tables S4, S5).

The reduction of the dataset to 5% of its original size led to significantly lower relative abundances of the majority of high and very high abundant species (Figure 2B and Supplementary Tables S4, S5). In addition, a misclassified species (*Pseudomonas* phage *Pf1*) with a relative abundance of 40% was identified (Figure 2B). This likely constitutes an artifact originated by the reduction of the size of the dataset.

Overall, these results demonstrate that sequencing depth has a major impact on the sensitivity of WMS for taxonomic profiling of samples with 90% host DNA. When decreasing sequencing depth, the number of microbial species that are not detected increase, along with unclassified and misclassified clades.

### Influence of the Level of Host DNA on the Sensitivity of WMS for Microbiome Taxonomic Profiling at a Fixed Sequencing Depth

**FIGURE 3:**
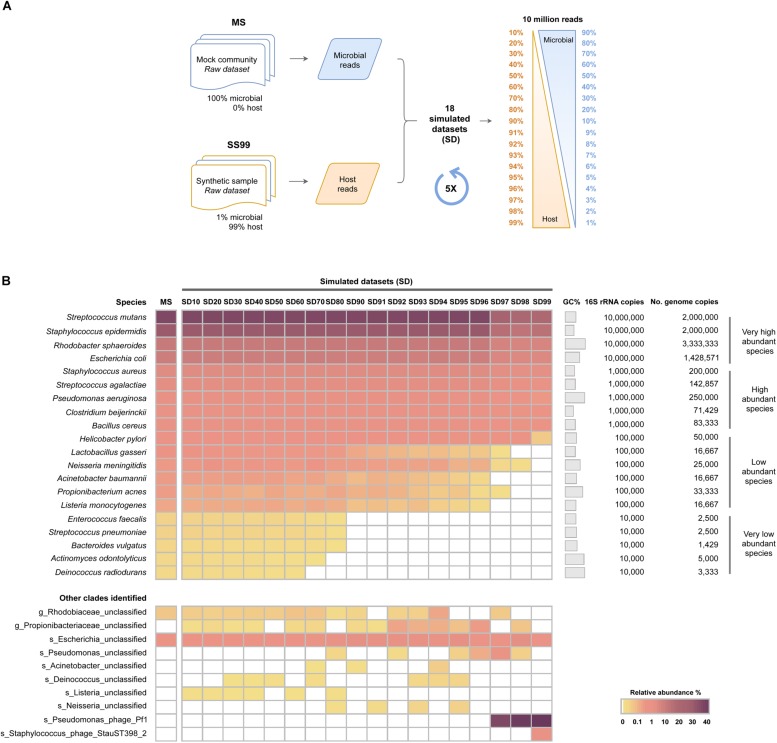
Influence of host DNA on the sensitivity of WMS for microbiome taxonomic profiling at a fixed sequencing depth. **(A)** Schematic representation of the experimental design to generate simulated datasets (SD) with different host: microbial ratios. Microbial and host single-end reads were randomly selected from the mock microbial community (MS) and from the SS99 raw datasets, and were combined in different proportions, at a fixed sequencing depth of 10 million reads, to generate 18 simulated datasets (SD) with progressively higher host reads (ranging from 10 to 99%). **(B)** Taxonomic profile of the SD are represented as the average relative abundance from five independent simulations, and shown in a heat map. Data was sorted from the highest to the lowest relative abundances of species in the mock microbial community (MS).

Having shown that high proportions of host DNA and reduced sequencing depths interfere with the sensitivity of WMS for microbiome profiling, the next aim was to investigate the influence of the level of host DNA on the sensitivity of the method at a fixed sequencing depth. For that, SD were generated with progressively greater proportions of host DNA (SD10 to SD99, ranging from 10 to 99% host reads), at the fixed sequencing depth of 10 million single-end reads with 150 bp length (1.5 Gb). This depth was chosen based on the recent guidelines for best practices for shotgun metagenomics, which suggest a minimum of 1 Gb sequencing depth per-sample (Quince et al., 2017). SD were composed of microbial and mouse reads, randomly picked from our previously generated MS and SS99 raw datasets, respectively (Figure 3A). For each of the SD, 5 replicates were generated, and the taxonomic profile was estimated with MetaPhlAn2, after sequencing data pre-processing, and compared to that of MS.

In SD10 to SD60, all 20 species of bacteria were successfully detected, without significant differences in their relative abundances in comparison with the MS control (Figure 3B and Supplementary Tables S6, S7). In SD70 to SD90, there was a progressive reduction of the number of very low abundant species identified, none of them being detected in SD90 (Figure 3B and Supplementary Tables S6, S7), a result in line with the random subsampling analysis performed above (Figure 2B). In SD92 to SD99, in addition to not identifying all of the very low abundant species, there was a statistically significant decrease in the relative abundance of low abundant species (Figure 3B and Supplementary Tables S6, S7). In particular, when host reads represented 97 to 99% of the datasets, the low abundant species were mostly undetected, and the relative abundance of the majority of high abundant species significantly decreased. Additionally, the *Pseudomonas* phage *Pf1* was again identified with high relative abundances in these highly complex datasets (Figure 3B). In the most complex dataset (SD99) a *Staphylococcus* phage *StauST398-2* was also identified.

Overall, these results show that at the fixed depth of 10 million reads, which is currently used in metagenomic studies, high levels of host DNA interfere with an accurate reconstitution of the microbiome profile.

## Discussion

Samples with a high amount of host DNA remains a major challenge in whole metagenome analysis, affecting the efficiency of microbiome profiling (Quince et al., 2017). Here, we show that high proportions of host DNA, reduce the sensitivity of WMS for microbiome profiling, in particular to detect very low and low abundant species of bacteria. It is plausible that high ratios of host: bacteria DNA reduce sequence coverage of the microbial genomes, hindering subsequent taxonomic analysis. This is consistent with previous studies addressing the issue of human DNA contamination on WMS detection of the malaria parasite in clinical samples (Auburn et al., 2011; Oyola et al., 2013). Although these reports were not focused on microbiome characterization, they showed that low levels of human DNA (≤30%) in blood samples, resulted in higher average *Plasmodium* genome coverage (Auburn et al., 2011), whereas clinical samples containing >80% human DNA, yielded a low number of reads assigned to *P. falciparum* genome (Oyola et al., 2013). In line with these observations, Hasan et al. (2016) found that by decreasing the human DNA background in a clinical sample, the sensitivity to detect microbial species was improved.

We also show that sequencing depth influences the sensitivity of microbiome profiling by WMS in samples that contain high levels of host DNA. The generation of SS90 datasets with reduced sequencing depths, resulted in gradually decreased capacity to accurately profile the microbiome. A reduction in sequencing depth from 51 million to 25 million reads already decreased WMS sensitivity, by preventing the identification of 60% of the species with very low abundance. In agreement with these findings, Jovel et al. (2016) showed that an increase in the size of the dataset leads to both an improvement of detection of microbial species and a more consistent estimation of their relative abundances. Moreover, in a metagenomic study of the fecal microbial community from beef cattle, the identification of new microbial taxa markedly improved with larger sequencing depths (Zaheer et al., 2018).

We also demonstrate that, besides preventing the identification of all species with very low abundance, a reduction in sequencing depth to five million reads additionally affected the relative abundance estimates of low abundant species. A depth as low as 2.5 million reads also resulted in major impairment in estimating the relative abundances of high and very high abundant species. In contrast with our findings, a recent study found no differences in the taxonomic profile of a mock community at divergent sequencing depths ranging from 0.1 to 7.5 single-end million reads (Walsh et al., 2018). These discrepancies may reflect the absence of host DNA in the mock sample analyzed in that study, as compared to the high levels of host DNA in our study samples (90%). Taken together, our data and that of others, suggest that similar sequencing depths have distinct effects on the sensitivity of WMS for taxonomic profiling, depending on the sample. In fact, our analysis of SD with 10 million reads indicated that the reconstitution of the microbiome profile becomes more inaccurate as the amount of host DNA in a sample progressively increases.

Interestingly, and based on this analysis, the outcomes of sequencing different types of host-derived samples, at a depth of 1.5 Gb per sample, can be extrapolated. For example, when sequencing a stool sample, the whole microbial community is expected to be accurately reconstituted, considering the low amount of host DNA in this type of sample [<10%; (Human Microbiome Project Consortium, 2012b)]. However, when sequencing samples like saliva, throat, buccal mucosa, and vaginal swabs (>90% host DNA), the detection of very low and low abundant species is expected to be impaired. This becomes more problematic in case of sequencing a tissue sample, as the detection of very low to high abundant species will be hampered, since this type of sample contains mostly human DNA (97 to 99% reads) and a low microbial biomass (Zhang et al., 2015). This also highlights another important aspect, which is the urgent need for effective host DNA depletion and/or microbial enrichment methods for whole metagenome analysis of tissue samples.

To the best of our knowledge, this is the first in-depth analysis demonstrating that greater proportions of host DNA, together with low sequencing depths, reduce the sensitivity of WMS for microbiome profiling. Therefore, the results of this study can assist in the design of WMS experiments, by highlighting the importance of sample type and sequencing depth when characterizing the microbiome.

## Data Availability

The raw sequencing data has been deposited at the NCBI Sequence Read Archive (PRJNA521492).

## Author Contributions

RF, L-JvD, CK, and CF conceptualized and designed the study. JP-M, AH, IP-R, and MW acquired the data. JP-M, RF, CK, and CF performed the data analysis and interpretation. JP-M, RF, and CF drafted the manuscript. All authors revised the manuscript for important intellectual content.

## Conflict of Interest Statement

AH, L-JvD, MW, and CK were employed by company DDL Diagnostic Laboratory, Rijswijk, Netherlands. The remaining authors declare that the research was conducted in the absence of any commercial or financial relationships that could be construed as a potential conflict of interest.

## References

[B1] Abubucker S. Segata N. Goll J. Schubert A. M. Izard J. Cantarel B. L. (2012). Metabolic reconstruction for metagenomic data and its application to the human microbiome. *PLoS Comput. Biol.* 8:e1002358. 10.1371/journal.pcbi.1002358 22719234 PMC3374609

[B2] Andrews S. (2016). *FastQC A Quality Control Tool for High Throughput Sequence Data.* Available at: http://www.bioinformatics.babraham.ac.uk/projects/fastqc/ (accessed March 8, 2016).

[B3] Auburn S. Campino S. Clark T. G. Djimde A. A. Zongo I. Pinches R. (2011). An effective method to purify *Plasmodium falciparum* DNA directly from clinical blood samples for whole genome high-throughput sequencing. *PLoS One* 6:e22213. 10.1371/journal.pone.0022213 21789235 PMC3138765

[B4] Bolger A. M. Lohse M. Usadel B. (2014). Trimmomatic: a flexible trimmer for Illumina sequence data. *Bioinformatics* 30 2114–2120. 10.1093/bioinformatics/btu170 24695404 PMC4103590

[B5] Frank D. N. St Amand A. L. Feldman R. A. Boedeker E. C. Harpaz N. Pace N. R. (2007). Molecular-phylogenetic characterization of microbial community imbalances in human inflammatory bowel diseases. *Proc Natl Acad Sci U.S.A.* 104 13780–13785. 10.1073/pnas.0706625104 17699621 PMC1959459

[B6] Gilbert J. A. Blaser M. J. Caporaso J. G. Jansson J. K. Lynch S. V. Knight R. (2018). Current understanding of the human microbiome. *Nat. Med.* 24 392–400. 10.1038/nm.4517 29634682 PMC7043356

[B7] Hasan M. R. Rawat A. Tang P. Jithesh P. V. Thomas E. Tan R. (2016). Depletion of human DNA in spiked clinical specimens for improvement of sensitivity of pathogen detection by next-generation sequencing. *J. Clin. Microbiol.* 54 919–927. 10.1128/JCM.03050-15 26763966 PMC4809942

[B8] Human Microbiome Jumpstart Reference Strains Consortium Nelson K. E. Weinstock G. M. Highlander S. K. Worley K. C. Creasy H. H. (2010). A catalog of reference genomes from the human microbiome. *Science* 328 994–999. 10.1126/science.1183605 20489017 PMC2940224

[B9] Human Microbiome Project Consortium (2012a). A framework for human microbiome research. *Nature* 486 215–221. 10.1038/nature11209 22699610 PMC3377744

[B10] Human Microbiome Project Consortium (2012b). Structure, function and diversity of the healthy human microbiome. *Nature* 486 207–214. 10.1038/nature11234 22699609 PMC3564958

[B11] Jovel J. Patterson J. Wang W. Hotte N. O’keefe S. Mitchel T. (2016). Characterization of the gut microbiome using 16s or shotgun metagenomics. *Front. Microbiol.* 7:459. 10.3389/fmicb.2016.00459 27148170 PMC4837688

[B12] Knight R. Jansson J. Field D. Fierer N. Desai N. Fuhrman J. A. (2012). Unlocking the potential of metagenomics through replicated experimental design. *Nat. Biotechnol.* 30 513–520. 10.1038/nbt.2235 22678395 PMC4902277

[B13] Kostic A. D. Chun E. Robertson L. Glickman J. N. Gallini C. A. Michaud M. (2013). *Fusobacterium nucleatum* potentiates intestinal tumorigenesis and modulates the tumor-immune microenvironment. *Cell Host Microbe* 14 207–215. 10.1016/j.chom.2013.07.007 23954159 PMC3772512

[B14] Langmead B. Salzberg S. L. (2012). Fast gapped-read alignment with Bowtie 2. *Nat. Methods* 9 357–359. 10.1038/nmeth.1923 22388286 PMC3322381

[B15] Ley R. E. Turnbaugh P. J. Klein S. Gordon J. I. (2006). Microbial ecology: human gut microbes associated with obesity. *Nature* 444 1022–1023. 17183309 10.1038/4441022a

[B16] Lloyd-Price J. Mahurkar A. Rahnavard G. Crabtree J. Orvis J. Hall A. B. (2017). Strains, functions and dynamics in the expanded human microbiome project. *Nature* 550 61–66. 10.1038/nature23889 28953883 PMC5831082

[B17] Marchesi J. R. Ravel J. (2015). The vocabulary of microbiome research: a proposal. *Microbiome* 3:31. 10.1186/s40168-015-0094-5 26229597 PMC4520061

[B18] Oyola S. O. Gu Y. Manske M. Otto T. D. O’brien J. Alcock D. (2013). Efficient depletion of host DNA contamination in malaria clinical sequencing. *J. Clin. Microbiol.* 51 745–751. 10.1128/JCM.02507-12 23224084 PMC3592063

[B19] Qin J. Li R. Raes J. Arumugam M. Burgdorf K. S. Manichanh C. (2010). A human gut microbial gene catalogue established by metagenomic sequencing. *Nature* 464 59–65. 10.1038/nature08821 20203603 PMC3779803

[B20] Quince C. Walker A. W. Simpson J. T. Loman N. J. Segata N. (2017). Shotgun metagenomics, from sampling to analysis. *Nat. Biotechnol.* 35 833–844. 10.1038/nbt.3935 28898207

[B21] Segata N. Waldron L. Ballarini A. Narasimhan V. Jousson O. Huttenhower C. (2012). Metagenomic microbial community profiling using unique clade-specific marker genes. *Nat. Methods* 9 811–814. 10.1038/nmeth.2066 22688413 PMC3443552

[B22] Truong D. T. Franzosa E. A. Tickle T. L. Scholz M. Weingart G. Pasolli E. (2015). MetaPhlAn2 for enhanced metagenomic taxonomic profiling. *Nat. Methods* 12 902–903. 10.1038/nmeth.3589 26418763

[B23] Truong D. T. Tett A. Pasolli E. Huttenhower C. Segata N. (2017). Microbial strain-level population structure and genetic diversity from metagenomes. *Genome Res.* 27 626–638. 10.1101/gr.216242.116 28167665 PMC5378180

[B24] Venter J. C. Remington K. Heidelberg J. F. Halpern A. L. Rusch D. Eisen J. A. (2004). Environmental genome shotgun sequencing of the Sargasso Sea. *Science* 304 66–74. 10.1126/science.1093857 15001713

[B25] Walsh A. M. Crispie F. O’sullivan O. Finnegan L. Claesson M. J. Cotter P. D. (2018). Species classifier choice is a key consideration when analysing low-complexity food microbiome data. *Microbiome* 6:50. 10.1186/s40168-018-0437-0 29554948 PMC5859664

[B26] Zaheer R. Noyes N. Ortega Polo R. Cook S. R. Marinier E. Van Domselaar G. (2018). Impact of sequencing depth on the characterization of the microbiome and resistome. *Sci. Rep.* 8:5890. 10.1038/s41598-018-24280-8 29651035 PMC5897366

[B27] Zhang C. Cleveland K. Schnoll-Sussman F. Mcclure B. Bigg M. Thakkar P. (2015). Identification of low abundance microbiome in clinical samples using whole genome sequencing. *Genome Biol.* 16:265. 10.1186/s13059-015-0821-z 26614063 PMC4661937

