# Supplementary material for: Impact of Host DNA and Sequencing Depth on the Taxonomic Resolution of Whole Metagenome Sequencing for Microbiome Analysis
Source: Front Microbiol. 2019 Jun 12;10:1277. doi: 10.3389/fmicb.2019.01277 (PMC6581681; doi:10.3389/fmicb.2019.01277)
Supplement: Supplementary file 1 [file Presentation_1.pdf]

*Supplementary Material*

# Impact of host DNA and sequencing depth on the taxonomic resolution of whole metagenome sequencing for microbiome analysis

**Joana Pereira-Marques[1,2,3], Anne Hout[4], Rui M. Ferreira[1,2], Michiel Weber[4], Ines Pinto-Ribeiro[1,2,5], Leen-Jan van Doorn[4], Cornelis Willem Knetsch[4,*], Ceu Figueiredo[1,2,5,*]**

[1]i3S – Instituto de Investigação e Inovação em Saúde, Universidade do Porto, Porto, Portugal;

[2]Ipatimup – Institute of Molecular Pathology and Immunology of the University of Porto, Porto, Portugal;

[3]Instituto de Ciências Biomédicas Abel Salazar da Universidade do Porto, Porto, Portugal;

[4]DDL Diagnostic Laboratory, Rijswijk, The Netherlands; and

[5]Faculty of Medicine of the University of Porto, Porto, Portugal.

*** Correspondence:**
Cornelis Willem Knetsch: Wilco.Knetsch@ddl.nl

Ceu Figueiredo:cfigueiredo@ipatimup.pt

**Supplementary Table S1.** Composition of the mock microbial community (B: HM-277D, Staggered, High Concentration, v5.2H), showing the expected and the observed relative abundance of species after WMS. The ratio of observed to expected relative abundance of species is also shown. Grey shading indicates a ≥ 2-fold change**.**

| Microbial species | NCBI assembly accession | 16S rRNA copies | 16S rRNA copies per genome [1] | No. genome copies | GC content [2] (%) | Expected relative abundance [3] (%) | Observed relative abundance (%) | Ratio observed/ expected |
|---|---|---|---|---|---|---|---|---|
| *Rhodobacter sphaeroides* ATCC 17023 | GCF_003324715.1 | 10,000,000 | 3 | 3,333,333 | 69 | 34.43 | 12.79 | 0.37 |
| *Streptococcus mutans* ATCC 700610 | GCF_000007465.2 | 10,000,000 | 5 | 2,000,000 | 37 | 20.66 | 36.21 | 1.75 |
| *Staphylococcus epidermidis* ATCC 12228 | GCF_000007645.1 | 10,000,000 | 5 | 2,000,000 | 32 | 20.66 | 27.10 | 1.31 |
| *Escherichia coli* ATCC 700926 | GCF_002843685.1 | 10,000,000 | 7 | 1,428,571 | 51 | 14.75 | 10.81 | 0.73 |
| *Pseudomonas aeruginosa* ATCC 47085 | GCF_000006765.1 | 1,000,000 | 4 | 250,000 | 67 | 2.58 | 1.47 | 0.57 |
| *Staphylococcus aureus* ATCC BAA-1717 | GCF_000017085.1 | 1,000,000 | 5 | 200,000 | 33 | 2.07 | 4.27 | 2.07 |
| *Streptococcus agalactiae* ATCC BAA-611 | GCF_000007265.1 | 1,000,000 | 7 | 142,857 | 36 | 1.48 | 2.80 | 1.90 |
| *Bacillus cereus* ATCC 10987 | GCF_000008005.1 | 1,000,000 | 12 | 83,333 | 36 | 0.86 | 0.99 | 1.14 |
| *Clostridium beijerinckii* ATCC 51743 | GCF_000016965.1 | 1,000,000 | 14 | 71,429 | 30 | 0.74 | 1.14 | 1.55 |
| *Helicobacter pylori* ATCC 700392 | GCF_000307795.1 | 100,000 | 2 | 50,000 | 39 | 0.52 | 0.81 | 1.58 |
| *Propionibacterium acnes* DSM 16379 | GCF_000008345.1 | 100,000 | 3 | 33,333 | 60 | 0.34 | 0.19 | 0.55 |
| *Neisseria meningitidis* ATCC BAA-335 | GCF_000008805.1 | 100,000 | 4 | 25,000 | 52 | 0.26 | 0.20 | 0.77 |
| *Acinetobacter baumannii* ATCC 17978 | GCF_001593425.2 | 100,000 | 6 | 16,667 | 39 | 0.17 | 0.19 | 1.13 |
| *Listeria monocytogenes* ATCC BAA-679 | GCF_000196035.1 | 100,000 | 6 | 16,667 | 38 | 0.17 | 0.16 | 0.93 |
| *Lactobacillus gasseri* ATCC 33323 | GCF_000014425.1 | 100,000 | 6 | 16,667 | 35 | 0.17 | 0.23 | 1.32 |
| *Actinomyces odontolyticus* ATCC 17982 | GCF_000154225.1 | 10,000 | 2 | 5,000 | 65 | 0.05 | 0.02 | 0.44 |
| *Deinococcus radiodurans* ATCC 13939 | GCF_001638825.1 | 10,000 | 3 | 3,333 | 67 | 0.03 | 0.02 | 0.56 |
| *Streptococcus pneumoniae* ATCC BAA-334 | GCF_000006885.1 | 10,000 | 4 | 2,500 | 40 | 0.03 | 0.04 | 1.55 |
| *Enterococcus faecalis* ATCC 47077 | GCF_000172575.2 | 10,000 | 4 | 2,500 | 38 | 0.03 | 0.04 | 1.57 |
| *Bacteroides vulgatus* ATCC 8482 | GCF_000012825.1 | 10,000 | 7 | 1,429 | 42 | 0.01 | 0.02 | 1.65 |

[1] Ribosomal RNA Database Curated by the Schmidt Laboratory (https://rrndb.umms.med.umich.edu/search/);
[2] NCBI genome database (https://www.ncbi.nlm.nih.gov/genome/genomes/714);
[3] Expected relative abundance = No. genome copies of each species/ sum of genome copies of all species.

**Supplementary Table S2.** Summary of the sequencing data pre-processing of synthetic samples (SS) metagenomes.

| Sample | Total number of raw single-end reads | Total number of quality-filtered reads | Total number of quality-filtered and host decontaminated reads |
|---|---|---|---|
| Microbial sample (MS) | 39,682,202 | 33,309,964 | 33,309,243 |
| Synthetic sample with 10% host DNA (SS10) | 40,087,736 | 32,489,550 | 29,899,628 |
| Synthetic sample with 90% host DNA (SS90) | 50,846,240 | 41,297,894 | 5,535,588 |
| Synthetic sample with 99% host DNA (SS99) | 36,098,546 | 30,214,162 | 746,018 |

**Supplementary Table S3.** Ratio of relative abundances of species from synthetic samples (SS) to MS. Grey shading indicates a ≥ 2-fold change**.**

| Microbial species | 16S rRNA copies | MS | SS10/MS | SS90/MS | SS99/MS |
|---|---|---|---|---|---|
| *Rhodobacter sphaeroides* ATCC 17023 | 10,000,000 | 1 | 0.805 | 0.814 | 1.053 |
| *Streptococcus mutans* ATCC 700610 | 10,000,000 | 1 | 1.081 | 1.061 | 0.877 |
| *Staphylococcus epidermidis* ATCC 12228 | 10,000,000 | 1 | 1.069 | 1.053 | 0.776 |
| *Escherichia coli* ATCC 700926 | 10,000,000 | 1 | 0.816 | 0.774 | 0.886 |
| *Pseudomonas aeruginosa* ATCC 47085 | 1,000,000 | 1 | 0.742 | 0.798 | 0.714 |
| *Staphylococcus aureus* ATCC BAA-1717 | 1,000,000 | 1 | 1.106 | 1.085 | 0.796 |
| *Streptococcus agalactiae* ATCC BAA-611 | 1,000,000 | 1 | 1.048 | 1.022 | 0.711 |
| *Bacillus cereus* ATCC 10987 | 1,000,000 | 1 | 1.021 | 1.237 | 0.574 |
| *Clostridium beijerinckii* ATCC 51743 | 1,000,000 | 1 | 1.064 | 1.070 | 0.589 |
| *Helicobacter pylori* ATCC 700392 | 100,000 | 1 | 0.911 | 0.907 | 0.561 |
| *Propionibacterium acnes* DSM 16379 | 100,000 | 1 | 0.625 | 0.875 | 0 |
| *Neisseria meningitidis* ATCC BAA-335 | 100,000 | 1 | 0.952 | 1.011 | 0.169 |
| *Acinetobacter baumannii* ATCC 17978 | 100,000 | 1 | 0.967 | 0.962 | 0 |
| *Listeria monocytogenes* ATCC BAA-679 | 100,000 | 1 | 0.943 | 0.824 | 0 |
| *Lactobacillus gasseri* ATCC 33323 | 100,000 | 1 | 1.091 | 0.910 | 0 |
| *Actinomyces odontolyticus* ATCC 17982 | 10,000 | 1 | 0.812 | 0.435 | 0 |
| *Deinococcus radiodurans* ATCC 13939 | 10,000 | 1 | 0.626 | 0 | 0 |
| *Streptococcus pneumoniae* ATCC BAA-334 | 10,000 | 1 | 1.006 | 0.608 | 0 |
| *Enterococcus faecalis* ATCC 47077 | 10,000 | 1 | 1.065 | 0.310 | 0 |
| *Bacteroides vulgatus* ATCC 8482 | 10,000 | 1 | 0.935 | 0.181 | 0 |

**Supplementary Table S4.** Ratio of relative abundances of species from each SS90 subset (SS90D50, SS90D25, SS90D10, SS90D5) to the SS90 original dataset (SS90D100). Random subsampling to generate each subset was performed in five independent experiments. Grey shading indicates a ≥ 2-fold change. nd: not detected in the SS90 original dataset.

| Microbial Species | 16S rRNA copies | SS90D100 | SS90D50/ SS90D100 | SS90D25/ SS90D100 | SS90D10/ SS90D100 | SS90D5/ SS90D100 |
|---|---|---|---|---|---|---|
| *Streptococcus mutans* ATCC 700610 | 10,000,000 | 1 | 1.004 | 0.999 | 1.002 | 0.625 |
| *Staphylococcus epidermidis* ATCC 12228 | 10,000,000 | 1 | 1.004 | 1.023 | 1.037 | 0.621 |
| *Rhodobacter sphaeroides* ATCC 17023 | 10,000,000 | 1 | 0.995 | 0.995 | 1.019 | 0.585 |
| *Escherichia coli* ATCC 700926 | 10,000,000 | 1 | 1.029 | 1.009 | 0.961 | 0.622 |
| *Staphylococcus aureus* ATCC BAA-1717 | 1,000,000 | 1 | 1.008 | 0.997 | 0.989 | 0.588 |
| *Streptococcus agalactiae* ATCC BAA-611 | 1,000,000 | 1 | 0.988 | 0.994 | 0.936 | 0.568 |
| *Pseudomonas aeruginosa* ATCC 47085 | 1,000,000 | 1 | 0.950 | 0.922 | 0.794 | 0.329 |
| *Clostridium beijerinckii* ATCC 51743 | 1,000,000 | 1 | 0.990 | 0.975 | 0.864 | 0.382 |
| *Bacillus cereus* ATCC 10987 | 1,000,000 | 1 | 1.000 | 0.950 | 0.971 | 0.465 |
| *Helicobacter pylori* ATCC 700392 | 100,000 | 1 | 0.957 | 0.964 | 0.776 | 0.257 |
| *Lactobacillus gasseri* ATCC 33323 | 100,000 | 1 | 0.949 | 0.677 | 0.105 | 0 |
| *Neisseria meningitidis* ATCC BAA-335 | 100,000 | 1 | 0.994 | 0.811 | 0.328 | 0 |
| *Acinetobacter baumannii* ATCC 17978 | 100,000 | 1 | 0.861 | 0.690 | 0.156 | 0 |
| *Propionibacterium acnes* DSM 16379 | 100,000 | 1 | 0.871 | 0.772 | 0.011 | 0 |
| *Listeria monocytogenes* ATCC BAA-679 | 100,000 | 1 | 0.850 | 0.483 | 0 | 0 |
| *Enterococcus faecalis* ATCC 47077 | 10,000 | 1 | 0.090 | 0 | 0 | 0 |
| *Streptococcus pneumoniae* ATCC BAA-334 | 10,000 | 1 | 0.271 | 0 | 0 | 0 |
| *Bacteroides vulgatus* ATCC 8482 | 10,000 | 1 | 0 | 0 | 0 | 0 |
| *Actinomyces odontolyticus* ATCC 17982 | 10,000 | 1 | 0 | 0 | 0 | 0 |
| *Deinococcus radiodurans* ATCC 13939 | 10,000 | nd | nd | nd | nd | nd |

**Supplementary Table S5**. Statistical analysis (*P*-values) of the results presented in Table S4. The Kruskal-Wallis non-parametric test followed by multiple comparisons (SS90D50, SS90D25, SS90D10, or SS90D5) versus a control group (SS90D100) using Dunn's test was performed for each species. NA: not applicable, as it was not detected in the SS90 original dataset.

| Microbial species | 16S rRNA copies | SS90D100 vs. SS90D50 | SS90D100 vs. SS90D25 | SS90D100 vs. SS90D10 | SS90D100 vs. SS90D5 |
|---|---|---|---|---|---|
| *Streptococcus mutans* ATCC 700610 | 10,000,000 | > 0.9999 | > 0.9999 | > 0.9999 | 0.0203 |
| *Staphylococcus epidermidis* ATCC 12228 | 10,000,000 | > 0.9999 | 0.2548 | 0.0341 | 0.7829 |
| *Rhodobacter sphaeroides* ATCC 17023 | 10,000,000 | > 0.9999 | > 0.9999 | > 0.9999 | 0.0102 |
| *Escherichia coli* ATCC 700926 | 10,000,000 | 0.9766 | > 0.9999 | > 0.9999 | 0.1242 |
| *Staphylococcus aureus* ATCC BAA-1717 | 1,000,000 | > 0.9999 | > 0.9999 | > 0.9999 | 0.0203 |
| *Streptococcus agalactiae* ATCC BAA-611 | 1,000,000 | 0.8729 | > 0.9999 | 0.0382 | 0.0004 |
| *Pseudomonas aeruginosa* ATCC 47085 | 1,000,000 | > 0.9999 | > 0.9999 | 0.0231 | 0.0004 |
| *Clostridium beijerinckii* ATCC 51743 | 1,000,000 | > 0.9999 | 0.6831 | 0.223 | 0.0009 |
| *Bacillus cereus* ATCC 10987 | 1,000,000 | > 0.9999 | > 0.9999 | > 0.9999 | 0.0048 |
| *Helicobacter pylori* ATCC 700392 | 100,000 | > 0.9999 | > 0.9999 | 0.0292 | 0.0006 |
| *Lactobacillus gasseri* ATCC 33323 | 100,000 | > 0.9999 | 0.1874 | 0.0025 | 0.0004 |
| *Neisseria meningitidis* ATCC BAA-335 | 100,000 | > 0.9999 | 0.7944 | 0.0242 | 0.0009 |
| *Acinetobacter baumannii* ATCC 17978 | 100,000 | 0.9033 | 0.2575 | 0.0024 | 0.0003 |
| *Propionibacterium acnes* DSM 16379 | 100,000 | > 0.9999 | 0.6463 | 0.0015 | 0.0015 |
| *Listeria monocytogenes* ATCC BAA-679 | 100,000 | > 0.9999 | 0.1586 | 0.0006 | 0.0006 |
| *Enterococcus faecalis* ATCC 47077 | 10,000 | 0.006 | 0.0007 | 0.0007 | 0.0007 |
| *Streptococcus pneumoniae* ATCC BAA-334 | 10,000 | 0.4097 | 0.0011 | 0.0011 | 0.0011 |
| *Bacteroides vulgatus* ATCC 8482 | 10,000 | 0.0005 | 0.0005 | 0.0005 | 0.0005 |
| *Actinomyces odontolyticus* ATCC 17982 | 10,000 | 0.0005 | 0.0005 | 0.0005 | 0.0005 |
| *Deinococcus radiodurans* ATCC 13939 | 10,000 | NA | NA | NA | NA |

**Supplementary Table S6**. Ratio of relative abundances of species from each simulated dataset (SD) to MS dataset. Grey shading indicates a ≥ 2-fold change. Random subsampling to generate each simulated dataset was performed in five independent experiments.

| Microbial Species | 16S rRNA copies | MS | SD10 /MS | SD20 /MS | SD30 /MS | SD40 /MS | SD50 /MS | SD60 /MS | SD70 /MS | SD80 /MS | SD90 /MS | SD91 /MS | SD92 /MS | SD93 /MS | SD94 /MS | SD95 /MS | SD96 /MS | SD97 /MS | SD98 /MS | SD99 /MS |
|---|---|---|---|---|---|---|---|---|---|---|---|---|---|---|---|---|---|---|---|---|
| *Streptococcus mutans* | 10,000,000 | 1 | 1.001 | 1.003 | 0.999 | 1.001 | 0.999 | 0.994 | 0.998 | 1.002 | 0.995 | 1.006 | 0.997 | 1.011 | 1.008 | 1.011 | 1.015 | 0.639 | 0.590 | 0.584 |
| *Staphylococcus epidermidis* | 10,000,000 | 1 | 1.001 | 1.005 | 1.003 | 1.005 | 1.005 | 1.006 | 1.002 | 1.005 | 1.015 | 1.002 | 1.003 | 1.027 | 1.013 | 1.005 | 1.038 | 0.659 | 0.572 | 0.603 |
| *Rhodobacter sphaeroides* | 10,000,000 | 1 | 1.002 | 0.993 | 0.996 | 1.000 | 1.001 | 1.001 | 0.998 | 0.994 | 0.988 | 1.000 | 1.005 | 0.985 | 1.001 | 0.983 | 1.005 | 0.638 | 0.570 | 0.572 |
| *Escherichia coli* | 10,000,000 | 1 | 1.003 | 1.014 | 0.997 | 0.987 | 0.974 | 0.987 | 1.008 | 0.960 | 0.913 | 0.946 | 0.965 | 0.911 | 0.982 | 0.959 | 0.940 | 0.597 | 0.520 | 0.499 |
| *Staphylococcus aureus* | 1,000,000 | 1 | 1.001 | 1.008 | 1.007 | 1.010 | 1.015 | 1.001 | 1.003 | 0.996 | 1.009 | 1.017 | 0.986 | 0.999 | 0.983 | 0.981 | 0.989 | 0.620 | 0.530 | 0.525 |
| *Streptococcus agalactiae* | 1,000,000 | 1 | 0.998 | 1.004 | 1.002 | 0.998 | 0.994 | 1.000 | 0.996 | 1.007 | 1.014 | 0.966 | 0.960 | 0.977 | 0.941 | 0.971 | 0.959 | 0.601 | 0.532 | 0.395 |
| *Pseudomonas aeruginosa* | 1,000,000 | 1 | 0.977 | 0.994 | 0.980 | 0.997 | 0.990 | 1.009 | 0.985 | 0.982 | 0.920 | 0.880 | 0.918 | 0.879 | 0.902 | 0.809 | 0.919 | 0.478 | 0.411 | 0.139 |
| *Clostridium beijerinckii* | 1,000,000 | 1 | 0.989 | 0.999 | 1.007 | 1.003 | 0.979 | 0.979 | 0.996 | 0.944 | 0.946 | 0.943 | 1.000 | 0.920 | 0.901 | 0.911 | 0.911 | 0.538 | 0.484 | 0.304 |
| *Bacillus cereus* | 1,000,000 | 1 | 1.039 | 0.977 | 1.037 | 0.978 | 1.036 | 1.023 | 0.991 | 1.003 | 0.903 | 0.979 | 1.065 | 0.997 | 1.011 | 1.015 | 0.851 | 0.698 | 0.568 | 0.435 |
| *Helicobacter pylori* | 100,000 | 1 | 0.992 | 0.982 | 0.990 | 0.990 | 0.955 | 0.963 | 0.939 | 0.923 | 0.909 | 0.953 | 0.967 | 0.851 | 0.806 | 0.827 | 0.798 | 0.435 | 0.301 | 0.072 |
| *Lactobacillus gasseri* | 100,000 | 1 | 0.941 | 0.939 | 0.919 | 0.924 | 0.910 | 0.903 | 0.906 | 0.890 | 0.698 | 0.588 | 0.509 | 0.413 | 0.424 | 0.305 | 0.295 | 0.033 | 0 | 0 |
| *Neisseria meningitidis* | 100,000 | 1 | 1.026 | 1.097 | 1.034 | 1.051 | 1.043 | 1.006 | 1.067 | 0.988 | 0.950 | 0.929 | 0.892 | 0.875 | 0.884 | 0.670 | 0.710 | 0.113 | 0.045 | 0 |
| *Acinetobacter baumannii* | 100,000 | 1 | 0.978 | 0.957 | 0.971 | 0.951 | 0.963 | 0.874 | 0.888 | 0.744 | 0.533 | 0.675 | 0.601 | 0.485 | 0.397 | 0.241 | 0.123 | 0 | 0 | 0 |
| *Propionibacterium acnes* | 100,000 | 1 | 0.780 | 0.830 | 0.749 | 0.674 | 0.843 | 0.760 | 0.712 | 0.858 | 0.676 | 0.736 | 0.659 | 0.460 | 0.320 | 0.381 | 0.078 | 0.034 | 0 | 0 |
| *Listeria monocytogenes* | 100,000 | 1 | 0.895 | 0.932 | 0.931 | 0.975 | 0.979 | 0.883 | 0.948 | 0.875 | 0.515 | 0.573 | 0.479 | 0.576 | 0.292 | 0.216 | 0.106 | 0 | 0 | 0 |
| *Enterococcus faecalis* | 10,000 | 1 | 0.858 | 0.820 | 0.837 | 0.705 | 0.820 | 0.588 | 0.325 | 0.210 | 0 | 0 | 0 | 0 | 0 | 0 | 0 | 0 | 0 | 0 |
| *Streptococcus pneumoniae* | 10,000 | 1 | 0.885 | 0.757 | 0.848 | 0.852 | 0.641 | 0.711 | 0.532 | 0.177 | 0 | 0 | 0 | 0 | 0 | 0 | 0 | 0 | 0 | 0 |
| *Bacteroides vulgatus* | 10,000 | 1 | 0.819 | 0.782 | 0.627 | 0.699 | 0.581 | 0.505 | 0.483 | 0.030 | 0 | 0 | 0 | 0 | 0 | 0 | 0 | 0 | 0 | 0 |
| *Actinomyces odontolyticus* | 10,000 | 1 | 0.778 | 0.655 | 0.685 | 0.538 | 0.319 | 0.157 | 0.015 | 0 | 0 | 0 | 0 | 0 | 0 | 0 | 0 | 0 | 0 | 0 |
| *Deinococcus radiodurans* | 10,000 | 1 | 0.704 | 0.831 | 0.662 | 0.523 | 0.428 | 0.212 | 0 | 0 | 0 | 0 | 0 | 0 | 0 | 0 | 0 | 0 | 0 | 0 |

**Supplementary Table S7.** Statistical analysis (*P*-values) of the results presented in Table S6. The Kruskal-Wallis non-parametric test followed by multiple comparisons (SD10, SD20, SD30, SD40, SD50, SD60, SD70, SD80, SD90, SD91, SD92, SD93, SD94, SD95, SD96, SD97, SD98 or SD99) versus a control group (MS) using Dunn's test was performed for each species.

| Microbial species | 16S rRNA copies | MS vs. SD10 | MS vs. SD20 | MS vs. SD30 | MS vs. SD40 | MS vs. SD50 | MS vs. SD60 | MS vs. SD70 | MS vs. SD80 | MS vs. SD90 | MS vs. SD91 | MS vs. SD92 | MS vs. SD93 | MS vs. SD94 | MS vs. SD95 | MS vs. SD96 | MS vs. SD97 | MS vs. SD98 | MS vs. SD99 |
|---|---|---|---|---|---|---|---|---|---|---|---|---|---|---|---|---|---|---|---|
| *Streptococcus mutans* | 10,000,000 | >0.999 | >0.999 | >0.999 | >0.999 | >0.999 | >0.999 | >0.999 | >0.999 | >0.999 | >0.999 | >0.999 | >0.999 | >0.999 | >0.999 | >0.999 | 0.172 | 0.2236 | 0.1963 |
| *Staphylococcus epidermidis* | 10,000,000 | >0.999 | >0.999 | >0.999 | >0.999 | >0.999 | >0.999 | >0.999 | >0.999 | >0.999 | >0.999 | >0.999 | 0.1454 | >0.999 | >0.999 | 0.1608 | >0.999 | >0.999 | >0.999 |
| *Rhodobacter sphaeroides* | 10,000,000 | >0.999 | >0.999 | >0.999 | >0.999 | >0.999 | >0.999 | >0.999 | >0.999 | >0.999 | >0.999 | >0.999 | >0.999 | >0.999 | >0.999 | >0.999 | 0.8504 | 0.1066 | 0.0994 |
| *Escherichia coli* | 10,000,000 | >0.999 | >0.999 | >0.999 | >0.999 | >0.999 | >0.999 | >0.999 | >0.999 | >0.999 | >0.999 | >0.999 | >0.999 | >0.999 | >0.999 | >0.999 | 0.0832 | 0.0195 | 0.0166 |
| *Staphylococcus aureus* | 1,000,000 | >0.999 | >0.999 | >0.999 | >0.999 | >0.999 | >0.999 | >0.999 | >0.999 | >0.999 | >0.999 | >0.999 | >0.999 | >0.999 | >0.999 | >0.999 | 0.6271 | 0.3169 | 0.3169 |
| *Streptococcus agalactiae* | 1,000,000 | >0.999 | >0.999 | >0.999 | >0.999 | >0.999 | >0.999 | >0.999 | >0.999 | >0.999 | >0.999 | >0.999 | >0.999 | 0.2796 | >0.999 | 0.6096 | 0.0412 | 0.018 | 0.0053 |
| *Pseudomonas aeruginosa* | 1,000,000 | >0.999 | >0.999 | >0.999 | >0.999 | >0.999 | >0.999 | >0.999 | >0.999 | 0.7215 | >0.999 | >0.999 | 0.3926 | >0.999 | 0.0747 | >0.999 | 0.0085 | 0.0038 | 0.0007 |
| *Clostridium beijerinckii* | 1,000,000 | >0.999 | >0.999 | >0.999 | >0.999 | >0.999 | >0.999 | >0.999 | >0.999 | >0.999 | >0.999 | >0.999 | 0.8975 | >0.999 | 0.5757 | >0.999 | 0.0153 | 0.0105 | 0.0028 |
| *Bacillus cereus* | 1,000,000 | >0.999 | >0.999 | >0.999 | >0.999 | >0.999 | >0.999 | >0.999 | >0.999 | >0.999 | >0.999 | >0.999 | >0.999 | >0.999 | >0.999 | >0.999 | 0.3371 | 0.4981 | 0.1312 |
| *Helicobacter pylori* | 100,000 | >0.999 | >0.999 | >0.999 | >0.999 | >0.999 | >0.999 | 0.8736 | >0.999 | 0.7417 | >0.999 | >0.999 | 0.9721 | 0.0462 | 0.2796 | 0.1899 | 0.0015 | 0.0005 | 0.0002 |
| *Lactobacillus gasseri* | 100,000 | >0.999 | >0.999 | >0.999 | >0.999 | >0.999 | >0.999 | >0.999 | >0.999 | 0.4485 | 0.0702 | 0.0371 | 0.012 | 0.0154 | 0.0044 | 0.0039 | 0.0001 | <0.0001 | <0.0001 |
| *Neisseria meningitidis* | 100,000 | >0.999 | >0.999 | >0.999 | >0.999 | >0.999 | >0.999 | >0.999 | >0.999 | >0.999 | >0.999 | >0.999 | >0.999 | >0.999 | >0.999 | >0.999 | 0.1467 | 0.1057 | 0.081 |
| *Acinetobacter baumannii* | 100,000 | >0.999 | >0.999 | >0.999 | >0.999 | >0.999 | >0.999 | >0.999 | 0.9099 | 0.1078 | 0.4353 | 0.1922 | 0.0841 | 0.0305 | 0.0078 | 0.0019 | 0.0002 | 0.0002 | 0.0002 |
| *Propionibacterium acnes* | 100,000 | >0.999 | >0.999 | >0.999 | >0.999 | >0.999 | >0.999 | >0.999 | >0.999 | >0.999 | >0.999 | 0.7519 | 0.0449 | 0.0154 | 0.0181 | 0.0004 | 0.0002 | 0.0001 | 0.0001 |
| *Listeria monocytogenes* | 100,000 | >0.999 | >0.999 | >0.999 | >0.999 | >0.999 | >0.999 | >0.999 | >0.999 | 0.1784 | 0.3442 | 0.1697 | 0.7687 | 0.0247 | 0.0123 | 0.0035 | 0.0005 | 0.0005 | 0.0005 |
| *Enterococcus faecalis* | 10,000 | >0.999 | >0.999 | >0.999 | >0.999 | >0.999 | >0.999 | 0.5228 | 0.1474 | 0.0007 | 0.0007 | 0.0007 | 0.0007 | 0.0007 | 0.0007 | 0.0007 | 0.0007 | 0.0007 | 0.0007 |
| *Streptococcus pneumoniae* | 10,000 | >0.999 | >0.999 | >0.999 | >0.999 | >0.999 | >0.999 | 0.7679 | 0.1013 | 0.0005 | 0.0005 | 0.0005 | 0.0005 | 0.0005 | 0.0005 | 0.0005 | 0.0005 | 0.0005 | 0.0005 |
| *Bacteroides vulgatus* | 10,000 | >0.999 | >0.999 | >0.999 | >0.999 | >0.999 | >0.999 | 0.9671 | 0.0035 | 0.0008 | 0.0008 | 0.0008 | 0.0008 | 0.0008 | 0.0008 | 0.0008 | 0.0008 | 0.0008 | 0.0008 |
| *Actinomyces odontolyticus* | 10,000 | >0.999 | >0.999 | >0.999 | >0.999 | >0.999 | >0.999 | 0.0036 | 0.0006 | 0.0006 | 0.0006 | 0.0006 | 0.0006 | 0.0006 | 0.0006 | 0.0006 | 0.0006 | 0.0006 | 0.0006 |
| *Deinococcus radiodurans* | 10,000 | >0.999 | >0.999 | >0.999 | >0.999 | >0.999 | 0.6434 | 0.0014 | 0.0014 | 0.0014 | 0.0014 | 0.0014 | 0.0014 | 0.0014 | 0.0014 | 0.0014 | 0.0014 | 0.0014 | 0.0014 |

<u>**Supplementary Figure S1.**</u>

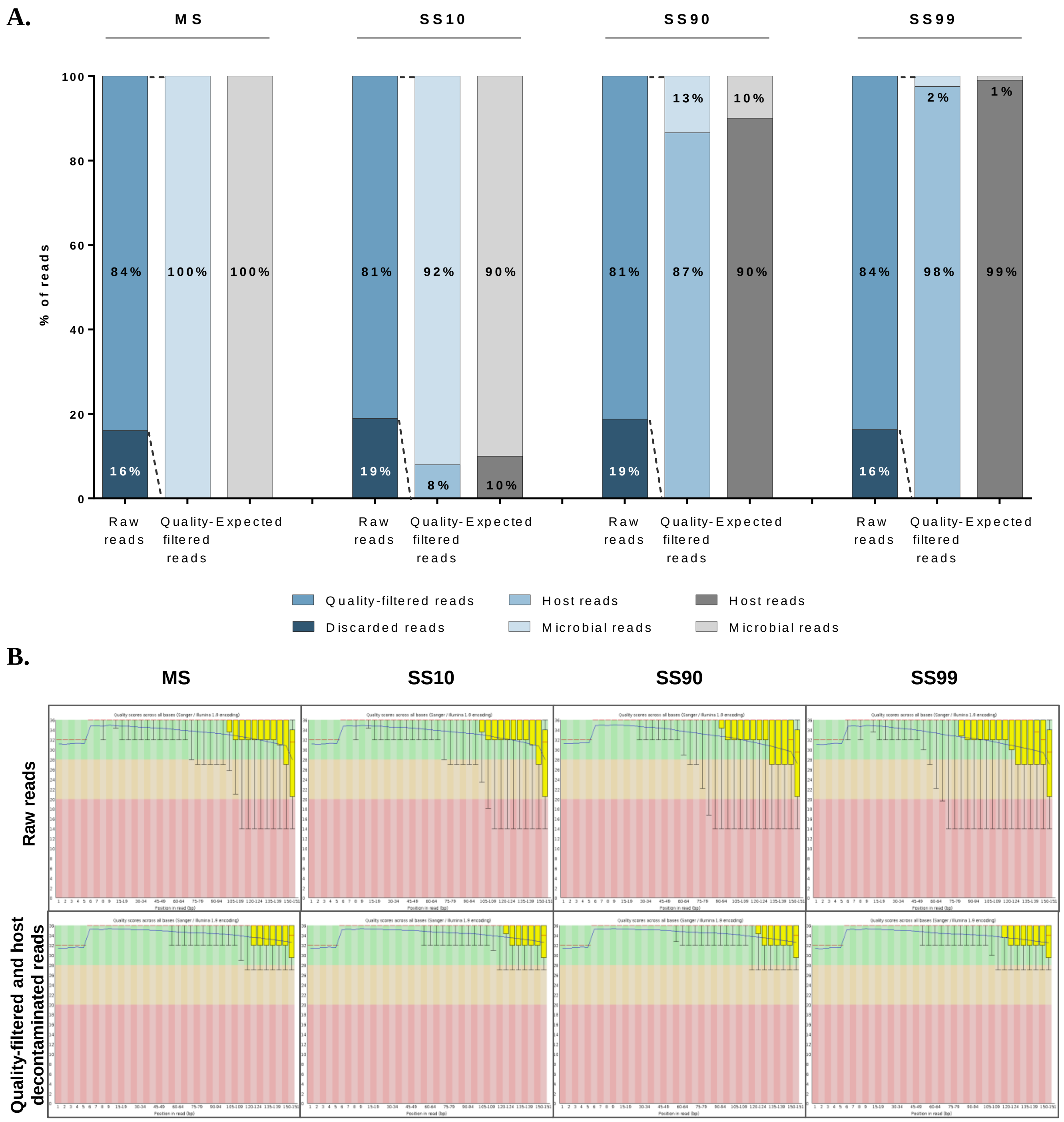


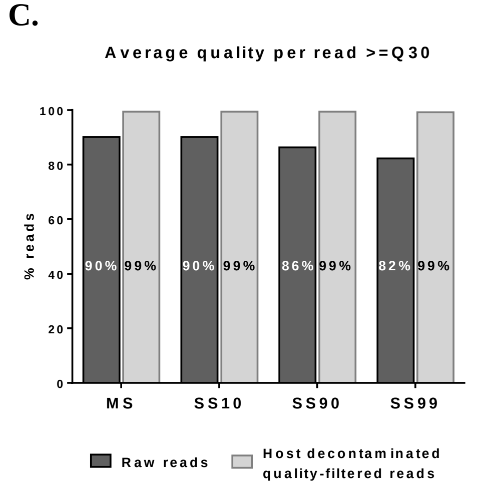


**Supplementary Figure S1. Overview of the sequencing data pre-processing from synthetic samples metagenomes. A.** Summary view of the fraction of quality-filtered, discarded, microbial and host reads per sample. The *raw reads* bar represents the percentage of quality-filtered and discarded reads upon Trimmomatic quality filtering. The *quality-filtered reads* bar constitutes the fraction of host and microbial quality-filtered reads obtained after performing both quality-filtering and host sequences decontamination steps. The *expected* bar consists in the theoretical percentages of host and microbial reads expected in each synthetic sample. **B.** FastQC graphs showing per base sequence quality in the raw sequence data and in the host decontaminated quality-filtered data from the MS, SS10, SS90 and SS99. Quality scores values across all bases at each position are shown. **C.** Percentage of reads with an average quality of ≥Q30 in the MS, SS10, SS90 and SS99, in the raw and host decontaminated quality-filtered data.

**Supplementary Figure S2.**

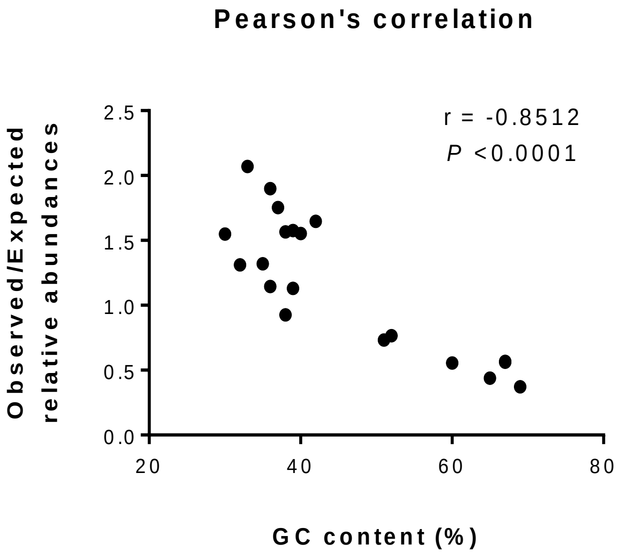


**Supplementary Figure S2. Correlation between genomic GC content and ratio of observed to expected relative abundances for the MS reference sample.** Pearson's correlation coefficient (r) and *P*-value for the association are shown in the graph. Since it has been shown that the GC content introduces a bias during Illumina Nextera XT library preparation and sequencing (Jones *et al.* 2015), this potential bias was evaluated in the MS dataset. Pearson's correlation analysis showed that the GC content was negatively correlated with the ratio of observed to expected relative abundances ($r = -0.8512$, $P < 0.0001$). This suggests that under and overestimated relative abundances were due to this bias.
